# Aryl Hydrocarbon Receptors in Osteoclast Lineage Cells Are a Negative Regulator of Bone Mass

**DOI:** 10.1371/journal.pone.0117112

**Published:** 2015-01-23

**Authors:** Tai-yong Yu, Wei-jun Pang, Gong-she Yang

**Affiliations:** 1 Division of Integrative Pathophysiology, Proteo-Science Center, Graduate School of Medicine, Ehime University, Ehime 791-0295, Japan; 2 Laboratory of Epigenetic Skeletal Diseases, Institute of Molecular and Cellular Biosciences, The University of Tokyo, Tokyo 113-0032, Japan; 3 College of Animal Science and Technology, Northwest A&F University, Yangling, Shaanxi Province, 712100, P.R. China; Inserm U606 and University Paris Diderot, FRANCE

## Abstract

Aryl hydrocarbon receptors (AhRs) play a critical role in various pathological and physiological processes. Although recent research has identified AhRs as a key contributor to bone metabolism following studies in systemic AhR knockout (KO) or transgenic mice, the cellular and molecular mechanism(s) in this process remain unclear. In this study, we explored the function of AhR in bone metabolism using *AhR^RANKΔOc/ΔOc^* (*RANK^Cre/+^;AhR^flox/flox^*) mice. We observed enhanced bone mass together with decreased resorption in both male and female 12 and 24-week-old *AhR^RANKΔOc/ΔOc^* mice. Control mice treated with 3-methylcholanthrene (3MC), an AhR agonist, exhibited decreased bone mass and increased bone resorption, whereas *AhR^CtskΔOc/ΔOc^* (*Ctsk^Cre/+^;AhR^flox/flox^*) mice injected with 3MC appeared to have a normal bone phenotype. *In vitro*, bone marrow-derived macrophages (BMDMs) from *AhR^RANKΔOc/ΔOc^* mice exhibited impaired osteoclastogenesis and repressed differentiation with downregulated expression of B lymphocyte-induced maturation protein 1 (*Blimp1*), and cytochrome P450 genes *Cyp1b1* and *Cyp1a2*. Collectively, our results not only demonstrated that AhR in osteoclast lineage cells is a physiologically relevant regulator of bone resorption, but also highlighted the need for further studies on the skeletal actions of AhR inhibitors in osteoclast lineage cells commonly associated with bone diseases, especially diseases linked to environmental pollutants known to induce bone loss.

## Introduction

Bone remodeling is a highly coordinated process that is primarily mediated by bone-forming osteoblasts and bone-resorbing osteoclasts [1–4]. However, functional dysregulation of osteoblasts and/or osteoclasts during the remodeling process can affect bone density and contribute to the pathogenesis of skeletal disorders [5, 6]. Diminished estrogen levels in women after menopause frequently promotes increased activation of osteoclastic bone resorption, which may be cause osteoporosis [7]. Osteoclastic bone resorption is also substantially elevated in inflammatory bone diseases such as rheumatoid arthritis [8]. Therefore, a better understanding of how the functions of differentiated early or mature osteoclasts are regulated in postnatal life is important to developing new and improved strategies for treating osteoporosis and inflammatory bone diseases.

The ligand-activated transcription factor AhR is a member of a transcription factor superfamily; it is characterized by structural motifs of basic helix-loop-helix (bHLH)/Per-AhR nuclear translocator (Arnt)-Sim (PAS) domains [9–11]. AhRs, or dioxin receptors, have a key role in inflammation and immune responses [12]. Recently, several groups have demonstrated that AhR regulates the balance of regulatory T cells and T helper (Th) type 17 (Th17) cells [13–15], providing evidence for the role of AhR in immune regulation. Skeletal homeostasis is dynamically influenced by the immune system [16–18]. Sato et al. examined the effect of various Th cell subsets on osteoclastogenesis and identified Th17 as an osteoclastogenic Th cell subset that links T cell activation and bone resorption [19]. AhR deficiency has been shown to ameliorate collagen-induced arthritis. Specifically, a lack of Ahr in T cells significantly suppressed collagen-induced arthritis development [20]. These findings indicated that AhR signaling may be associated with pathological and physiological processes of bone metabolism. More recently, two research groups reported that AhR is essential for the maintenance of bone mass [21, 22]; this was elucidated via the use of genetically engineered mouse models with either a systemic loss-of-function or gain-of-function of AhR. These results might be attributable to systemic alterations of the AhR gene in mice, which may cause secondary effects in other tissues and/or organs. Although the roles of AhR in bone homeostasis have been described, the direct function of AhRs in osteoclasts remains understood.

In this study, we use conditional knockout (KO) mouse models and show that bone mass increases accompanied by decreases in bone resorption occur in *AhR^RANKΔOc/ΔOc^* mice and *AhR^CtskΔOc/ΔOc^* mice. *AhR^CtskΔOc/ΔOc^* mice are tolerant to the bone loss typically induced by the AhR-agonist 3-methylcholanthrene (3MC). We provide physiological evidence of the influential role AhRs play in osteoclast lineage cells and subsequent bone metabolism. Furthermore, we suggest that inhibition of AhRs in osteoclast lineage cells may be beneficial in the treatment of osteoporosis and inflammatory bone diseases.

## Results

### Generation of the osteoclastic AhR deletion in mice

**Fig 1:**
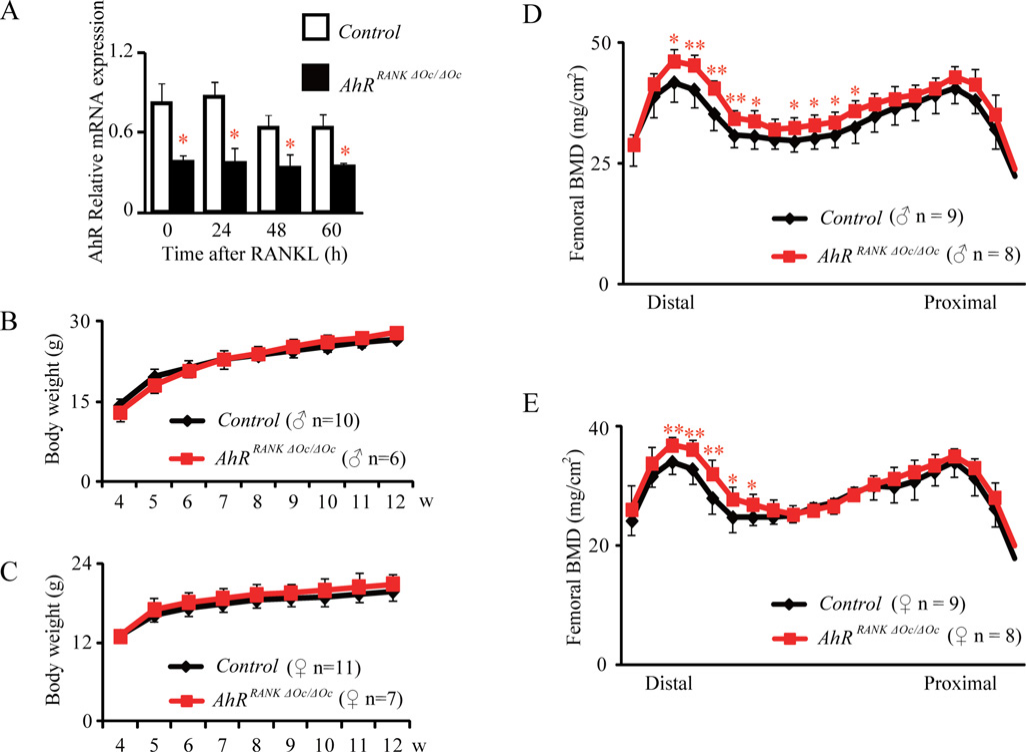
Osteoclast precusor AhR KO mice show increased bone mass. (A) Quantitative real-time PCR analysis of AhRs in primary cultured osteoclasts. BMDMs from 8-week-old *AhR* ^*RANK ΔOc/ΔOc*^ mice were cultured in the presence of RANKL. Cells were harvested at 0, 24, 48 and 60 h, and AhR levels were measured by qPCR. (B and C) Body weight was measured in 4 to 12-week-old male (B) and female (C) mice. DEXA measurement of whole femurs from 12-week-old *AhR* ^*RANKΔOc/ΔOc*^ mice and their littermate controls. BMD was assessed for 20 equal cross-sectional divisions of femurs (D and E).

To investigate the function of AhRs in osteoclasts, we generated mice lacking AhRs in osteoclasts by crossing AhR-floxed mice with *RANK*-*Cre* (also known as *Tnfrsf11a-Cre*) mice, a knockin mouse line expressing the Cre recombinase in osteoclast precursors. Quantitative real-time PCR analysis demonstrated that we had effectively deleted AhR in the osteoclasts of these newly generated mice, to yield mice with osteoclast precursors AhR deletion (*AhR^RANKΔOc/ΔOc^: RANK^Cre/+^;AhR^flox/flox^*) (Fig. 1A). Body weight was measured in 4 to 12-week-old mice. There was no significant difference in body weight between *AhR^RANKΔOc/ΔOc^* mice and their littermate controls (Fig. 1B–C).

### Mice with the osteoclastic AhR deletion exhibited an increased bone mass

The BMD of 12-week-old *AhR^RANKΔOc/ΔOc^* mice was measured by DEXA, which showed the distal femoral BMD of both male and female *AhR^RANKΔOc/ΔOc^* mice was significantly increased compared with littermate controls (Fig. 1D–E).

**Fig 2:**
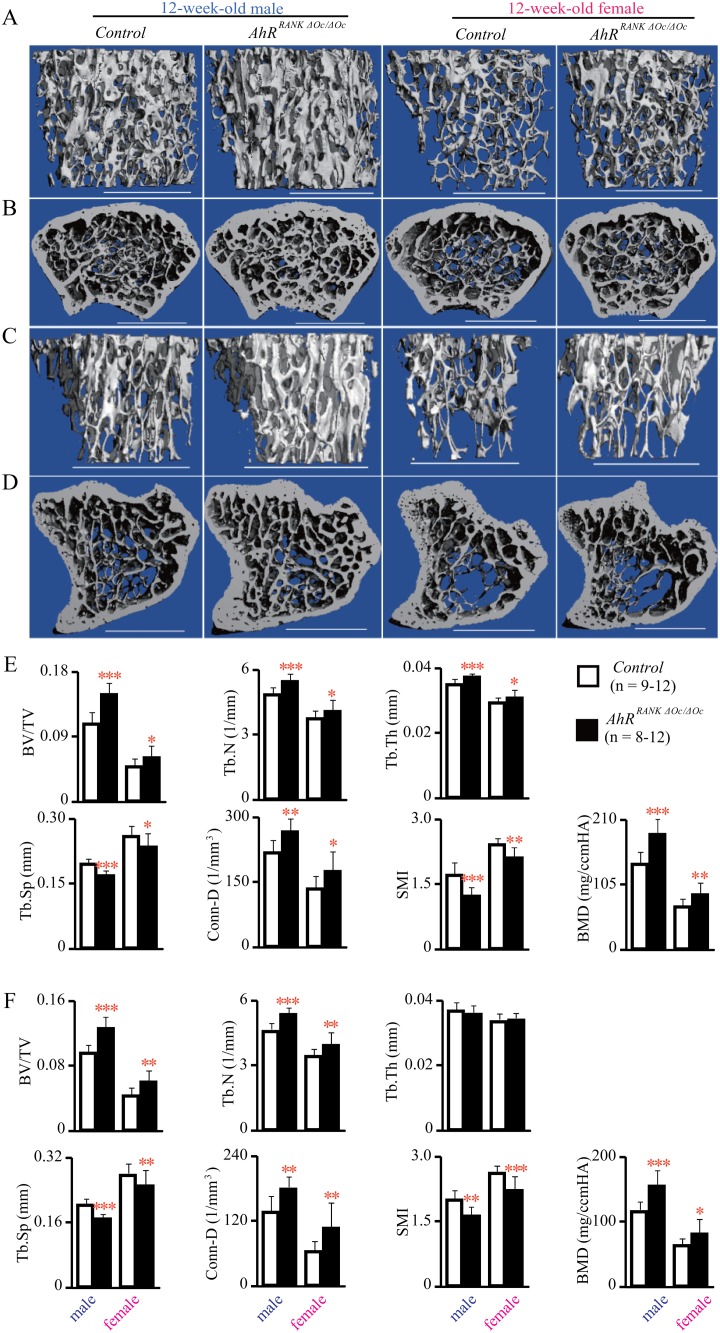
*μ*CT analyses of femurs and tibiaes in osteoclast precusor AhR KO mice. Representative images of trabecular bone in the distal femurs from 12-week-old *AhR^RANKΔOc/ΔOc^* mice (A) and axial sections of the distal metaphysis (B). Representative images of trabecular bone in the proximal tibiaes from 12-week-old *AhR^RANKΔOc/ΔOc^* mice (C) and axial sections of the proximal metaphysis (D). Scale bars indicate 1.0 mm. *μ*CT parameters of the distal femurs are shown in (E). *μ*CT parameters of the proximal tibiaes are shown in (F).

To assess changes in the three-dimensional trabecular architecture between *AhR^RANKΔOc/ΔOc^* mice and their littermate controls, *µ*CT was performed. Increased trabecular bone mass at the distal femur in *AhR^RANKΔOc/ΔOc^* mice was observed using *µ*CT analysis (Fig. 2A and 2B). The trabecular bone of both male and female *AhR^RANKΔOc/ΔOc^* mice exhibited a significant increase compared to those of their littermate controls in the following structural parameters: BV/TV, Tb.N, Tb.Th, BMD, and Conn-D; whereas a decrease versus controls was evident for Tb.Sp and SMI (Fig. 2E). In addition, this phenotype also was observed in the proximal tibiae of *AhR^RANKΔOc/ΔOc^* mice (Fig. 2C–D and F).

**Fig 3:**
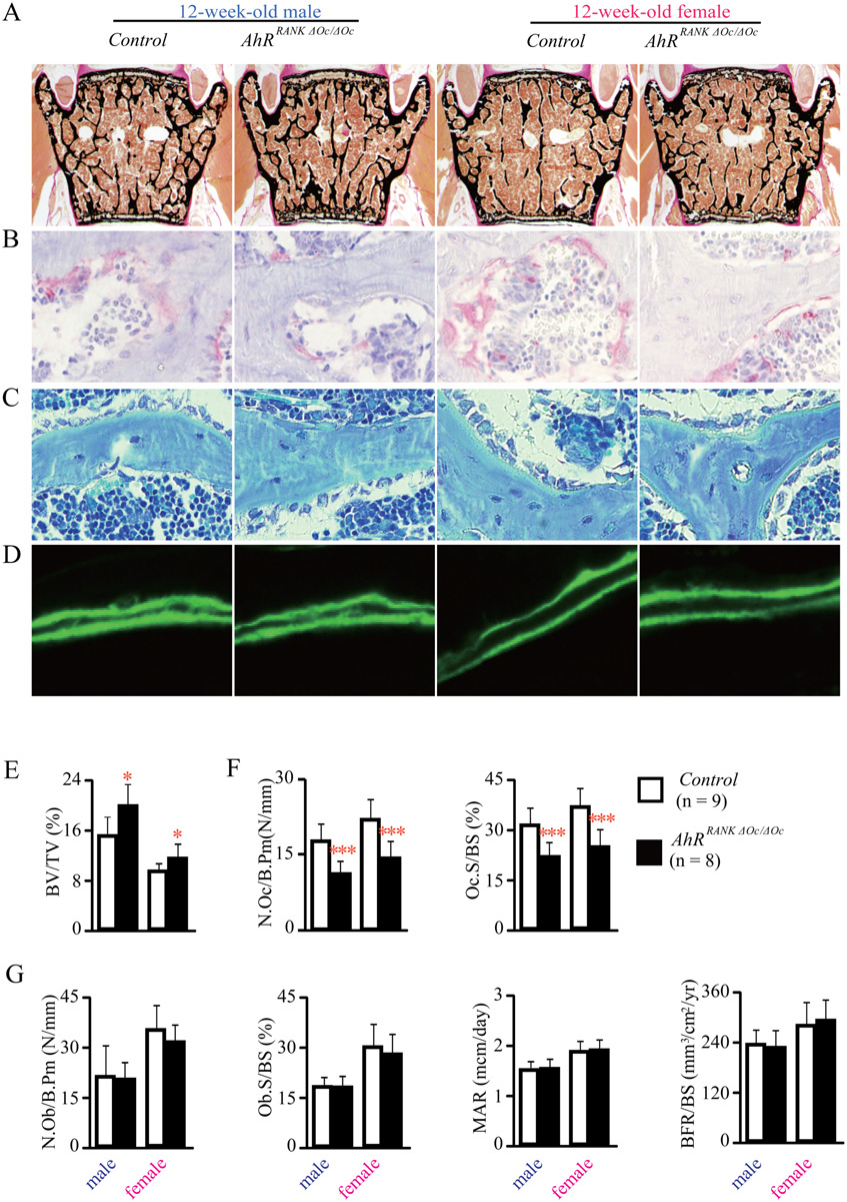
Osteoclast precusor AhR KO mice exhibit decreased bone resorption. Bone histomorphometric analysis of lumbar vertebrae from 12-week-old *AhR^RANKΔOc/ΔOc^* mice and their littermate controls. Sections were stained with von Kossa/van Gieson stain (A), TRAP stain (B), toluidine blue (C) or left unstained to evaluate calcein labeling (D). Trabecular bone volume (E), osteoclast number and surface (F), osteoblast number and surface, and dynamic histomorphometric variables (G) are shown.

To examine mineralized bone volume in the vertebral trabecular bone of *AhR^RANKΔOc/ΔOc^* mice, we performed von Kossa/van Gieson staining. Consistent with the femur and tibiae, histological analysis of vertebrae also demonstrated that both male and female *AhR^RANKΔOc/ΔOc^* mice displayed significantly greater vertebral BV/TV (Fig. 3A and E).

**Fig 4:**
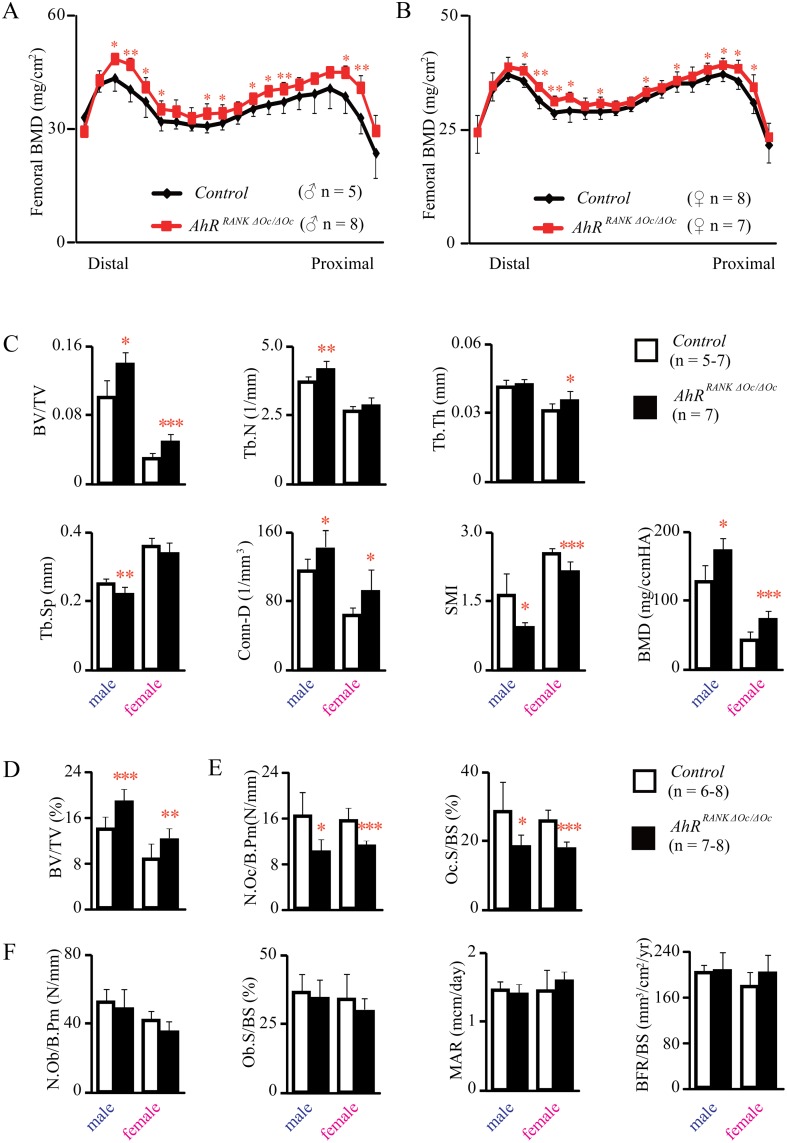
Analyses for bone phenotypes in 24-week-old osteoclast precusor AhR KO mice (*AhR^RANKΔOc/ΔOc^*). DEXA analyses of whole femurs. BMD was assessed for 20 equal cross-sectional divisions of femurs from 24-week-old *AhR^RANKΔOc/ΔOc^* mice and their littermate controls (A and B). Analyses of *μ*CT parameters for distal femurs (C). Bone histomorphometric analyses of lumbar vertebrae for trabecular bone volume (D), osteoclast number and surface (E), osteoblast number and surface, and dynamic histomorphometric parameters (F) are shown.

Given the effects of age, the bone phenotype of 24-week-old *AhR^RANKΔOc/ΔOc^* mice was evaluated by DEXA, *µ*CT, and von Kossa/van Gieson staining, and likewise showed dramatically increased bone mass when compared with the littermate controls (Fig. 4A–D).

### AhRs in osteoclasts regulate bone resorption

To define the cellular basis of the increased bone mass phenotype observed in *AhR^RANKΔOc/ΔOc^* mice, bone histomorphometry was performed. The number and/or activity of osteoblasts/osteoclasts were examined in the lumbar vertebrae, at L3 and L4, of 12-week-old *AhR^RANKΔOc/ΔOc^* mice and their littermate controls. Parameters related to osteoclastic bone resorption, such as N.Oc/B.Pm and Oc.S/BS, were significantly decreased in both male and female *AhR^RANKΔOc/ΔOc^* mice compared to their littermate controls (Fig. 3B and F). On the other hand, parameters related to osteoblastic bone formation, such as N.Ob/B.Pm, Ob.S/BS, MAR and BFR/BS, were not altered in *AhR^RANKΔOc/ΔOc^* mice (Fig. 3C–D and G). This phenotype also was observed in both male and female 24-week-old *AhR^RANKΔOc/ΔOc^* mice (Fig. 4E–F).

Another murine line with an osteoclastic-specific AhR deletion (*AhR^CtskΔOc/ΔOc^*: *Ctsk^Cre/+^;AhR^flox/flox^*) was created using *Cathepsin K* (*Ctsk*)-*Cre* mice (*Ctsk^Cre/+^*); this knockin mouse line expresses the Cre recombinase specifically in differentiated mature osteoclasts. These mice display similarities in terms of an increased bone mass together with reduced bone resorption (S1 Fig.) [23].

Taken together, the phenotypic results of *AhR^RANKΔOc/ΔOc^* and *AhR^CtskΔOc/ΔOc^* mice suggest that the presence of AhR-deficient osteoclasts could decrease the number of osteoclasts and consequently their bone resorbing activity, indicating that the increase in bone mass in mice with an AhR-deletion in osteoclasts could be the result of reduced osteoclastic bone resorption, rather than an increase in bone formation by osteoblasts.

### Bone loss triggered by the AhR-agonist 3MC is blocked in mice with an osteoclastic AhR deletion

An osteoclast-specific AhR deletion in mice was characterized by increased bone mass together with decreased bone resorption. If AhRs in osteoclasts are essential for bone remodeling, an AhR agonist should be able to enhance bone resorption and therefore diminish bone mass. To test this possibility, male mice with the osteoclastic AhR deletion (*AhR^CtskΔOc/ΔOc^*) and control mice were treated with 3MC, a well-characterized AhR agonist.

**Fig 5:**
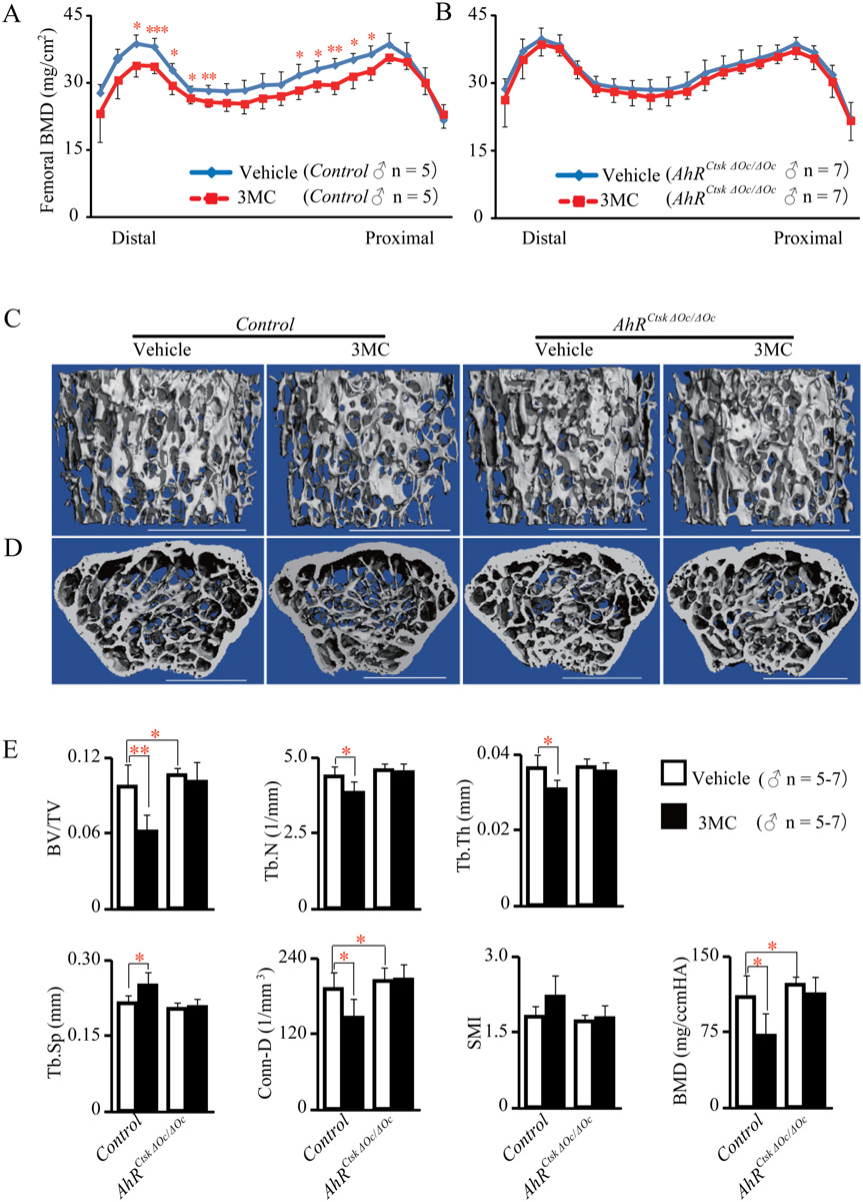
AhR agonist, 3MC, did not reduce bone mass in osteoclastic AhR KO mice. DEXA analyses of whole femurs. BMD was assessed for 20 equal cross-sectional divisions of femurs from 12-week-old *AhR^CtskΔOc/ΔOc^* male mice and their littermate controls treated with or without 3MC (A and B). *μ*CT images for analysis of femurs representative of trabecular bone in the distal femurs (C) and axial sections in the distal metaphysis (D). Scale bars indicate 1.0 mm. parameters of the distal femurs are shown in (E).

The BMD of whole femurs was measured by DEXA, and the results demonstrated that the BMD of 3MC-treated control mice was significantly decreased, compared with that of vehicle-treated controls (Fig. 5A). However, the BMD of femurs from 3MC-treated *AhR^CtskΔOc/ΔOc^* mice were not significantly different from those of vehicle-treated *AhR^CtskΔOc/ΔOc^* mice (Fig. 5B).

Bone structure at the distal femurs of mice was measured using *µ*CT. The results showed that treatment with 3MC induced an osteopenic phenotype in control mice by decreasing BV/TV, Tb.N, Tb.Th, Conn.D, and BMD, while increasing Tb.Sp compared with vehicle-treated controls. However, bone mass and structure of the *AhR^CtskΔOc/ΔOc^* mice was not impaired following 3MC treatment (Fig. 5C–E).

**Fig 6:**
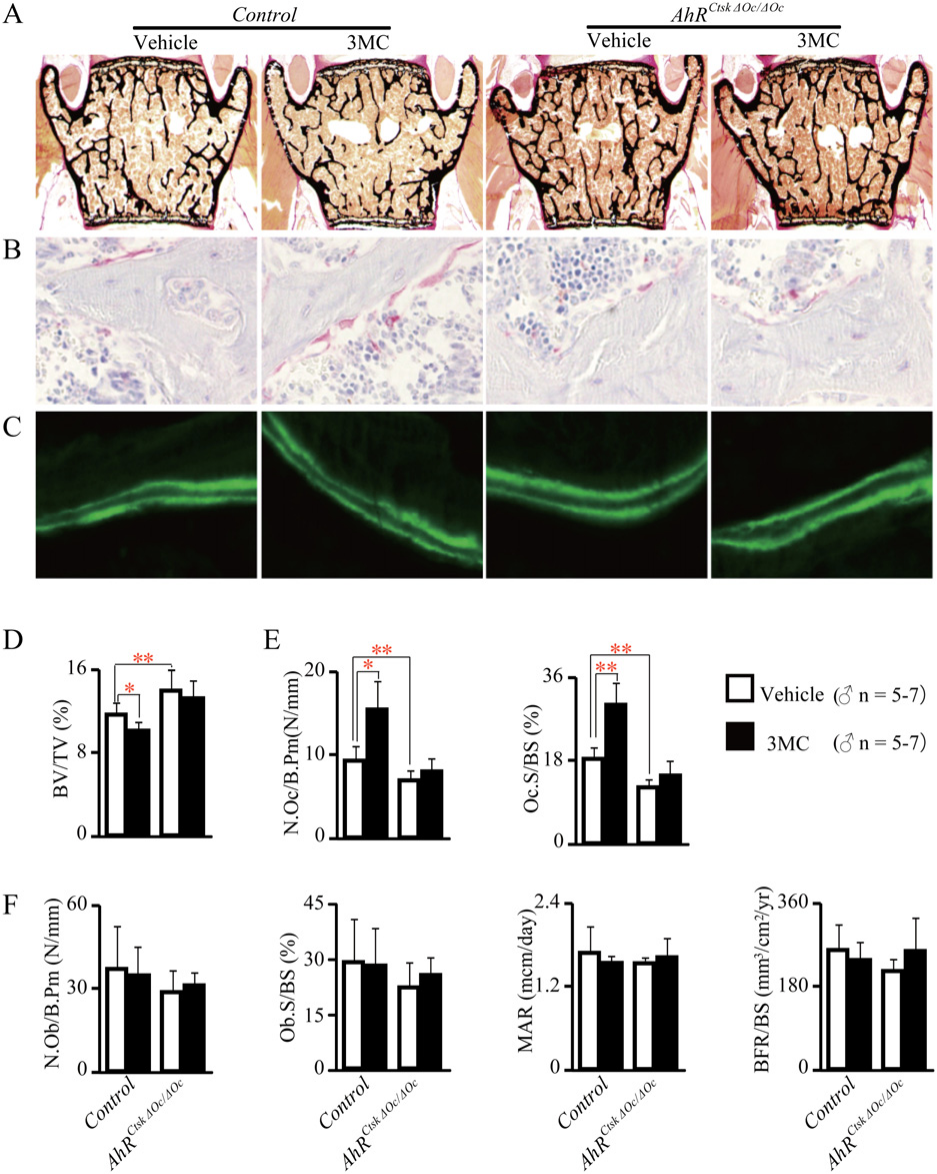
AhR agonist, 3MC, did not increase bone resorption in osteoclastic AhR KO mice. Bone histomorphometric analysis of lumbar vertebrae from 12-week-old *AhR^CtskΔOc/ΔOc^* male mice and their littermate controls treated with or without 3MC. Sections were stained with von Kossa/von Gieson stain (A), TRAP stain (B), or left unstained to evaluate calcein labeling (C). Trabecular bone volume (D), osteoclast number and surface (E), osteoblast number and surface, and dynamic histomorphometric parameters (F) are shown.

Bone histomorphometric analysis of lumbar vertebrae was performed, and the results showed that 3MC-induced bone loss was characterized by an increase in bone resorption in control mice, but not in *AhR^CtskΔOc/ΔOc^* mice (Fig. 6).

The same phenotype was also observed in *AhR^CtskΔOc/ΔOc^* female mice and their littermate controls treated with or without 3MC (data not shown).

### Impaired osteoclastogenesis and inhibited osteoclast differentiation in mice with an osteoclastic AhR deletion

**Fig 7:**
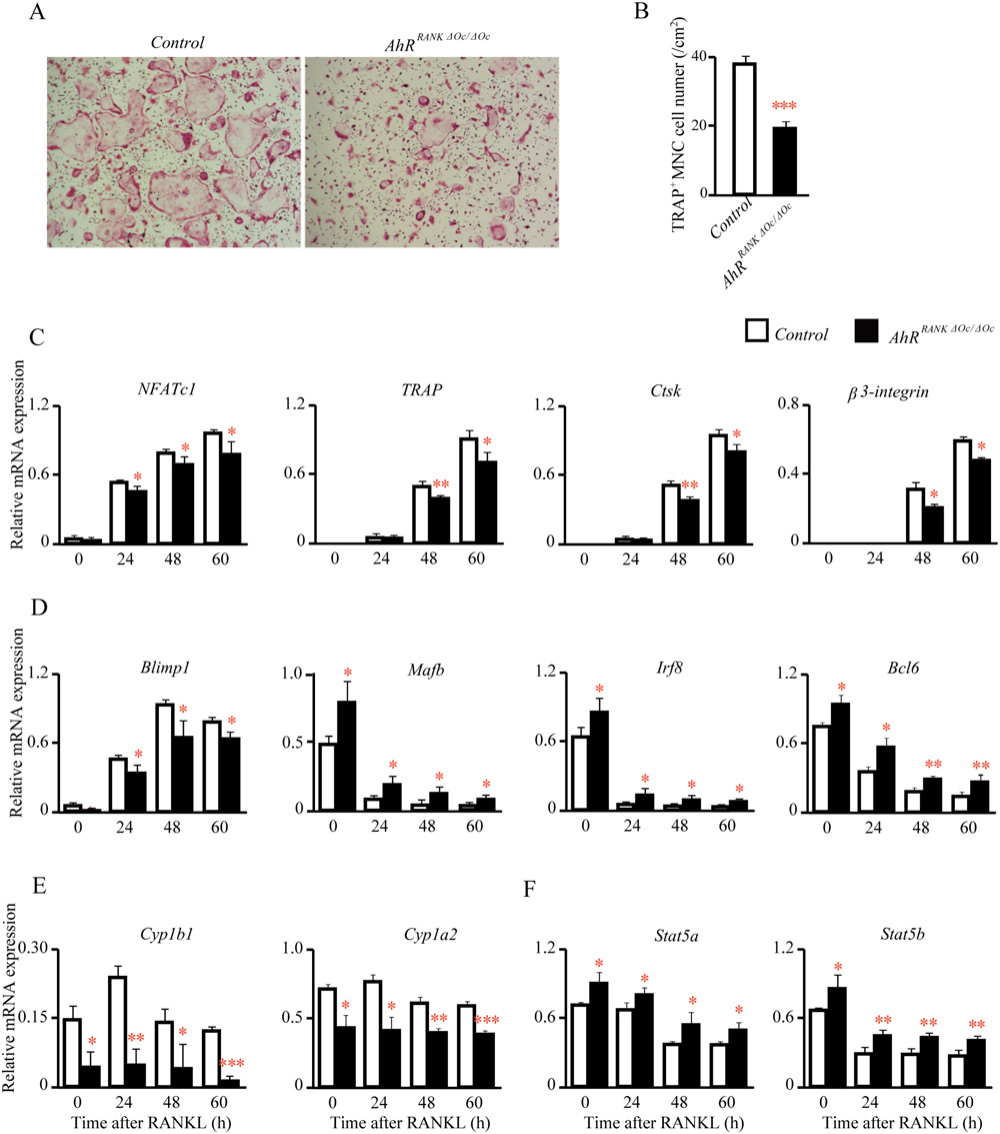
Impaired multinucleation in *AhR^RANKΔOc/ΔOc^* mice osteoclasts. (A) BMDMs from 8-week-old *AhR^RANKΔOc/ΔOc^* mice (*RANK^Cre/+^; AhR^flox/flox^*) and their littermate controls (*RANK^Cre/+^; AhR^+/+^*) were cultured in the presence of RANKL for 60 h, and cultures were stained for TRAP^+^ cells. (B) BMDMs from 8-week-old *AhR^RANKΔOc/ΔOc^* mice and their littermate controls were cultured in the presence of RANKL for 60 h, and the TRAP^+^ cells multinucleated cells (MNCs) containing more than three nuclei were counted as osteoclasts. (C) The expression levels of osteoclast differentiation markers were lower in *AhR^RANKΔOc/ΔOc^* osteoclasts. BMDMs from 8-week-old *AhR^RANKΔOc/ΔOc^* mice were cultured in the presence of RANKL. Cells were harvested at 0, 24, 48, and 60 h, and *NFATc1*, *TRAP*, *Ctsk*, *β3-integrin* levels were measured by qPCR. (D-F) QPCR analysis of *Blimp1*, *Mafb*, *Irf8*, *Cyp1b1*, *Cyp1a2*, *Stat5a*, and *Stat5b* levels in controls and *AhR^RANKΔOc/ΔOc^* BMDMs stimulated with RANKL for the indicated times. Females, n = 3 for controls and n = 4 for *AhR^RANKΔOc/ΔOc^*.

To evaluate the role of AhR in osteoclastogenesis, we evaluated osteoclastogenesis *in vitro* using BMDMs from *AhR^RANKΔOc/ΔOc^* and control (*RANK^Cre/+^; AhR^+/+^*) mice. We treated BMDMs with RANK ligand (RANKL), and TRAP-positive multinucleated cells as mature osteoclasts. RANKL-induced osteoclastogenesis was impaired (Fig. 7A), and TRAP-positive multinuclear cell (MNC) numbers were reduced in *AhR^RANKΔOc/ΔOc^* cells, compared with those in control cells (Fig. 7B). To check whether differentiation in *AhR^RANKΔOc/ΔOc^* osteoclasts was abnormal, we examined the expression levels of various osteoclast differentiation marker genes using quantitative real-time PCR. The expression levels of nuclear factor activated T-cells (*NFATc1*), *TRAP*, *Ctsk*, and *β3-integrin* were lower in *AhR^RANKΔOc/ΔOc^* osteoclasts (Fig. 7C).

### Deletion of AhR in osteoclasts disrupts osteoclastic gene expression

To further characterize how an AhR deletion in osteoclasts results in impaired osteoclastogenesis and reduced differentiation, expression levels of B lymphocyte-induced maturation protein 1 (*Blimp1*) and two cytochrome P450 genes (*Cyp1b1* and *Cyp1a2*), which are targets of AhR, were examined. Quantitative real-time PCR analysis showed that *Blimp1*, *Cyp1b1*, and *Cyp1a2* expression was downregulated in the absence of AhR in osteoclasts (Fig. 7D–E). In contrast, expression levels of musculoaponeurotic fibrosarcoma oncogene family protein B (*Mafb*), interferon regulatory factor-8 (*Ifr8*), and B-cell lymphoma 6 (*Bcl6*), which are negative target genes of Blimp1, were dramatically upregulated in *AhR^RANKΔOc/ΔOc^* osteoclasts (Fig. 7D). Additionally, we also examined the expression levels of AhR-related genes, including two members of the Signal Transducers and Activators of Transcription (*Stat*) family (*Stat5a* and *Stat5b*) and found they were also upregulated in AhR-deficient osteoclasts, compared with those in controls (Fig. 7F). These results indicate the absence of AhRs in osteoclasts leads to the invocation of several potential osteoclastogenesis and differentiation pathways.

## Discussion

Osteoclasts, exclusive bone resorbing cells, play pivotal roles in bone remodeling. Functional dysregulation of osteoclasts can cause skeletal diseases, such as osteoporosis [5, 7], osteopetrosis [6], and rheumatoid arthritis [24]. Obtaining a better understanding of osteoclast regulation is therefore central to the development of new treatments for bone disorders. It is well known that osteoclast differentiation requires macrophage colony-stimulating factor (M-CSF) and RANKL [25–27]. When these cytokines stimulate their receptors, *c-fms* and *RANK*, respectively, transcription factors such as the cellular oncogene fos (*c-Fos*) [28], nuclear factor kappa B (*NF-kB*) [29], and *NFATc1* [30] in osteoclast precursors are activated, and osteoclast differentiation is induced by the induction of osteoclastic genes such as *TRAP* and *Ctsk*. Additionally, extracellularly regulated kinase 1 (*Erk1*) [31], *Ly49Q* [32], *Wnt5α* [33], *Wnt5b* [34], tumor necrosis factor-alpha (*TNF-α*) [35, 36], and lipopolysaccharide (LPS) [37] positively regulate osteoclast differentiation, whereas osteoprotegrin (*OPG*) [38], *ERKs* [39], interferon gamma (*IFN-γ*) [40], *Irf-8* [41], *MafB* [42], *Bcl6* [43] inhibit generation of osteoclasts. Although many molecules have been described as regulators of osteoclast biology, the complex signaling networks that govern this process suggest that many more regulators of osteoclastogenesis remain to be identified. In this study, osteoclastic AhR KO mice exhibit increased bone mass together with decreased bone resorption *in vivo*, and impaired osteoclastogenesis and inhibited differentiation has been observed in murine cells with the AhR deletion *in vitro*. These results suggest that AhR is a novel positive regulator of function and differentiation in osteoclasts.

In Fig. 1D, 4A, 4B and S1 Fig., not only dital metaphysis but also diaphyseal BMD was increased in femur in mice with an AhR-deletion in osteoclasts. In addition, in Fig. 2E and 4C, *μ*CT analysis of the distal femurs demonstrated that not only BV/TV but also Tb.Th was increased in mice with an AhR-deletion in osteoclasts. It is known that diaphyseal BMD is influenced by bone formation and Tb.Th is regarded as a bone formation parameter, therefore, these results raises a possibility that bone formation may also be influenced in mice with an AhR-deletion in osteoclasts. Nevertheless, In Figs. 3C-D, 3G, 4F, bone histomorphometric analysis of the lumbar vertebrae demonstrated that N.Ob/B.Pm, Ob.S/BS, MAR and BFR/BS were not increased in mice with an AhR-deletion in osteoclasts. As we have studied before, Tb.Th, MAR and BFR/BS were not increased in systemic AhR knockout mice [23]. In particular, BMD, BV/TV, Tb.Th, N.Ob/B.Pm, Ob.S/BS, MAR and BFR/BS were all normal, failing to increase in osteoblastic AhR knockout mice [23]. Therefore, bone formation is not influenced by AhR.

*Cyp1* and *Blimp1* have been recognized as transcriptional target genes of AhR [21, 44]. Recently, Iqbal, et al. reported increased bone mass and low levels of osteoclastogenesis in *Cyp1a1/1a2/1b1-/-* triple KO mice [21]. Additionally, osteoclast-specific Blimp1 KO mice exhibited increased bone mass together with decreased bone resorption, via inhibited expression of *Ifr8*, *MafB*, and *Bcl6* [43, 45]. It is known that Ifr8 regulates bone metabolism by suppressing osteoclastogenesis [41], MafB negatively regulates RANKL-mediated osteoclast differentiation [42], and Bcl6 KO mice exhibit decreased bone mass with increased osteoclastogenesis [43]. In our study, *Blimp1* and *Cyp1* were downregulated in the absence of AhR in osteoclasts, whereas Mafb, Ifr8, and Bcl6 were dramatically upregulated (Fig. 7D–E). These results suggest that the ability of AhR in osteoclasts to regulate bone mass may be mediated by expression of one of its target genes, particularly *Blimp1*. Moreover, it is reported that bone resorption is regulated by the cell-autonomous negative feedback loop of the Stat5-Dusp axis in osteoclasts [46]. *Stat5a* and *Stat5b* also were upregulated in the absence of AhR in osteoclasts (Fig. 7F). This result suggests that in terms of bone metabolism, functional regulation of AhR in osteoclasts may also be related to suppressing the expression of Stat5 signaling.

It has been considered that exposure to environmental pollutants is a source of extreme physiological stress, potentially leading to a wide range of deleterious effects including skeletal diseases, endocrine disruption, immunosuppression, cancer, and developmental and reproductive defects [47]. Dioxins are persistent environmental pollutants, and their lipophilic content makes these compounds very resistant to degradation. Based on *in vivo* experiments, acute and short-term exposure to dioxins and dioxin-like compounds can cause profound effects in bone tissue, including geometrical changes, decreased bone strength, and decreased bone mineral density [48–51]. However, the mechanisms underlying these changes in bone are not fully understood. AhRs have been characterized as ligand-activated transcriptional factors induced in response to dioxins, such as 2,3,7,8-tetrachlorodibenzo-p-dioxin (TCDD) and 3MC. Most, if not all, deleterious effects of dioxins are mediated by AhR. Very few studies have addressed the mechanisms of bone toxicities caused by dioxins, making it a topic that should be investigated further. In the present study, we examined control mice treated with 3MC and showed that decreased bone mass together with increased bone resorption occurred, but in mice with an osteoclastic AhR deletion, injecting 3MC resulted in a normal bone phenotype (Figs. 5–6). Our study provide evidence that the toxic effects of AhR ligands, such as dioxin, are directly mediated by osteoclastic AhR and support the results of previous studies useing systemic AhR null mice [22]. This finding could help explain the unexpectedly profound bone loss and high fracture risk seen as a result of environmental pollutants exposure.

Overall, we have provided evidence showing that mice with an osteoclastic AhR deletion exhibit an increased bone mass together with decreased bone resorption, making them tolerant to bone loss induced by 3MC. Our studies are important and were designed to facilitate an understanding of the functional regulation of osteoclasts and the bone toxicity mechanisms resulting from environmental pollutants. Further research on osteoclastic AhR antagonists should be able to help provide more information on the development of tools that could be useful in the prevention and treatment of osteoporosis caused by environmental pollutants.

## Materials and Methods

### Mice

AhR floxed mice were provided by Dr. Y Fujii-Kuriyama. The Receptor Activator of Nuclear Factor-Kappa B (*RANK*)-*Cre* mice (*RANK^Cre/+^*) (also known as *Tnfrsf11a-Cre*), were kindly provided by Dr. Y. Kobayashi. Osteoclastic AhR KO mice (*AhR^RANKΔOc/ΔOc^: RANK^Cre/+^*;*AhR^flox/flox^*) were generated by breeding *RANK* ^Cre/Cre^;*AhR^flox/+^* male mice and *RANK* ^+/+^;*AhR^flox/+^* female mice, and *RANK* ^Cre/+^;*AhR^+/+^* were considered as controls. All mice were housed in a specific-pathogen-free (SPF) facility under climate-controlled conditions with a 12-hour light/dark cycle and were provided with water and standard diet (CE-2, CLEA, Japan) *ad libitum*. They were euthanized at 12 or 24 weeks of age. All animals were maintained and examined according to the protocol approved by the Animal Care and Use Committee of the University of Tokyo and Ehime University.

### Dual energy x-ray absorptiometry (DEXA) and Micro-computed tomography (*μ*CT) analysis

The bone mineral density (BMD) of total femurs was measured by DEXA using a bone mineral analyzer (DCS-600EX: ALOKA). Trabecular bone architecture of distal femurs was performed by using a *μ*CT system (*µ*CT35, SCANCO Medical, Bruttisellen, Switzerland). Bone specimens were stabilized with Styrofoam strips in a 6-ml tube filled with 70% ethanol and fastened in the specimen holder of the *μ*CT scanner. A 2.8 mm region was scanned with a spatial resolution of 6 μm, 70 kVp and 114 μA. 466 slices were acquired, starting just beneath the end of the growth plate, thus including both the primary and secondary spongiosa. From these scans, a region 1.8 mm in length of the distal metaphyseal secondary spongiosa (300 slices) was selected for analysis. Images were reconstructed into three-dimensional (3-D) volumes with the region of interest segmented using a fixed threshold. Unbiased, 3-D microstructural properties of trabecular bone, including bone volume over tissue volume (BV/TV), trabecular number (Tb.N), trabecular thickness (Tb.Th), trabecular separation (Tb.Sp), connectivity density (Conn.D, the trabecular number per mm^3^), structure model index (SMI, a measure of how plate- or rod-like trabeculaes are, 0 for parallel plates, 3 for cylindrical rods), and BMD were then calculated for the trabecular region of the metaphysis of the distal femur using methods based on distance transformation of the binarized images [52]. *μ*CT in tibiaes referred to the mentioned above, while it was the proximal part in tibiaes that was analyzed.

### Bone histomorphometric analysis

Bone histomorphometry was performed as previously described [53, 54]. In brief, lumbar vertebrae were dissected, fixed for 24 h in 4% paraformaldehyde (PFA) in phosphate-buffered saline (PBS), dehydrated in a series of graded ethanols, and embedded in methyl methacrylate (MMA) resin according to standard protocols. The plastic sections were cut by motorized rotary microtome (RM2255, LEICA, Germany) into 7 µm or 4 µm thick sections. Seven micron thick sections were stained with Von Kossa/Van Gieson stain to measure bone volume (BV/TV). To analyze osteoblasts (N.Ob/B.Pm: osteoblast number per bone perimeter; Ob.S/BS: osteoblast surface per bone surface) and osteoclasts (N.Oc/B.Pm: osteoclast number per bone perimeter; Oc.S/BS: osteoclast surface per bone surface), 4 μm thick sections were stained with toluidine blue and tartrate-resistant acid phosphatase (TRAP), respectively. Mineral apposition rate (MAR) and bone formation rate (BFR/BS) were analyzed on sections with double calcein labels. Calcein (Sigma-C0875–5G, USA) was dissolved in calcein buffer (0.15 M NaCl and 2% NaHCO3) and injected twice at 0.25 mg/g body weight on days 1 and 4, and then mice were euthanized and analyzed on day 6. For MAR and BFR/BS measurements, 7 μm thick sections were cleared in 1-Acetoxy- 2-Methoxyethane (AME, Wako, Japan). Bone histomorphometric analyses were performed using the OsteoMeasure analysis system (OsteoMetrics Inc., GA, USA) according to ASBMR guidelines [55].

### AhR-ligand treatment

Male control (*AhR^+/+^*: *Ctsk* ^Cre/+^ *;AhR^+/+^*) and osteoclastic AhR KO (*AhR^CtskΔOc/ΔOc^: Ctsk* ^Cre/+^;*AhR^flox/flox^*) littermates were treated with 3-Methylcholanthrene (3MC, Wako, Japan) or a placebo of corn oil starting when they were eight weeks old. 3MC was dissolved in corn oil and injected at 0.01 mg/g body weight, twice a week for four weeks. Mice were analyzed at 12 weeks of age.

### Osteoclast culture

Osteoclastogenesis *in vitro* was studied by plating bone marrow-derived macrophages (BMDMs) from 8-week-old mice in culture dishes containing α-MEM (GIBCO) with 10% FBS (MP Biomedicals). After incubation for 8 hr, nonadherent cells were collected, and cells were seeded (3×10^5^ cells/dish) in 6 cm suspension dishes containing α-MEM with 10 ng/ml M-CSF (R&D Systems). After 2 days (about 80% confluent), adherent cells were used as osteoclast precursor cells after washing out the nonadherent cells. Cells were cultured in the presence of 10 ng/ml M-CSF and 235 ng/ml GST-RANKL (Oriental yeast, Japan) for 60 hr to generate osteoclast like cells.

### Quantitative real-time PCR analysis

Total RNA was isolated with TRIzol reagent (Invitrogen) according to the manufacturer’s protocol. First-strand cDNA was synthesized from total RNA using PrimeScript RT Master Mix (Takara Bio Inc) and subjected to real-time PCR using KAPA SYBR Fast qPCR Kits (Kapa Biosystems) with Thermal Cycler Dice (Takara Bio Inc) according to the manufacturers’ instructions. Expression levels were normalized by *Gapdh*. The following primers were used: *Gapdh*, 5′-AAATGGTGAAGGTCGGTGTG-3′ and 5′-TGAAGGGGTCGTTGATGG-3′; *AhR*, 5′-TTCTATGCTTCCTCCACTATCCA-3′ and 5′-GGCTTCGTCCACTCCTTGT; *NFATC1*, 5′-TCGGTAGCCAGCCAGGAATC and 5′-GGGACCAACCGTATTTCCACAC-3′; *TRAP*, 5′-TCAGATCCATAGTGAAACCGCAAG-3′ and 5′-TTGCGACCATTGTTAGCCACATA-3′; *Ctsk*, 5′-CTTTGCCGTGGCGTTATACATACA-3′ and 5′-CAGCAGAACGGAGGCATTGA-3′; *β3-integrin*, 5′-CGCCTCGTGTGGTACAGAT-3′ and 5′-AGTGGCCGGGACAACTCT-3′; *Blimp1*, 5′-TGCTTATCCCAGCACCCC-3′ and 5′-CTTCAGGTTGGAGAGCTGACC-3′; *Mafb*, 5′-AACGGTAGTGTGGAGGAC-3′ and 5′-TCACAGAAAGAACTCAGGA-3′; *Irf8*, 5′-AAGGTCACCGTGGTCCTTAG-3′ and 5′-GGAAAGCCTTACCTGCTGAC-3′; *Bcl6*, 5′-AGACGCACAGTGACAAACCATACAA-3′ and 5′-GCTCCACAAATGTTACAGCGATAGG-3′; *Cyp1a2*, 5′-AAAGGGGTCTTTCCACTGCT-3′ and 5′-AGGGACACCTCACTGAATGG-3′; *Cyp1b1*, 5′-CACAACCTGGTCCAACTCAG-3′ and 5′-AGCCAGGACACCCTTTCC-3′; *Stat5a*, 5′-CCGAAACCTCTGGAATCTGA-3′ and 5′-ACGAACTCAGGGACCACTTG-3′; *Stat5b*, 5′-GTGAAGCCACAGATCAAGCA-3′ and 5′-TCGGTATCAAGGACGGAGTC-3′.

### Statistical analysis

Data were analyzed by a two-tailed student’s t-test. For all graphs, data are represented as mean ± SD. A *p*-value less than 0.05 was considered statistically significant (*p < 0.05; **p < 0.01; ***p < 0.001).

## Supporting Information

S1 Fig DEXA analyses of whole femurs in 12-week-old osteoclast AhR KO mice (*AhR^CtskΔOc/ΔOc^*). BMD was assessed for 20 equal cross-sectional divisions of femurs from 12-week-old *AhR^CtskΔOc/ΔOc^* mice and their littermate controls.
(EPS)
